# Trends and Hotspots in Nursing Theory Research Published from 1990 to 2022: A Web of Science-Based Bibliometric Analysis

**DOI:** 10.1155/2024/8667809

**Published:** 2024-08-17

**Authors:** Ruifang Zhu, Qian Wang, Jun Zhang, Weiliang Liu, Caizhen Ma, Shifan Han, Zhiguang Duan

**Affiliations:** ^1^ Editorial Office First Hospital of Shanxi Medical University, Taiyuan 030001, Shanxi, China; ^2^ School of Nursing Shanxi Medical University, Taiyuan 030001, Shanxi, China

## Abstract

**Methods:**

This study adopted bibliometric approaches and analyzed the nursing theory research literature included in the Web of Science database during the 33 years between 1990 and 2022 using VOSviewer software. The source countries and regions, subject categories and distributions of institutions/units, journals, and highly productive authors of 23,180 nursing theory publications from the past 33 years were analyzed. The top ten funding agencies of nursing theory literature were also analyzed. Cluster and topic evolution analyses were performed on high-frequency keywords in nursing theory literature.

**Results:**

Historical trends in nursing theory were explored based on the number of articles published each year. The authoritative academic journals of nursing theory were identified based on the number of articles published, and the leaders of nursing theory and their academic research teams were identified based on the authors and their institutions. Moreover, the research hotspots and development trends in nursing theory were explored based on keyword clustering and topic evolution analysis. Thus, it is helpful to guide nursing practice and effectively solve nursing problems in nursing management.

**Conclusion:**

Research on nursing theory has been increasingly applied in clinical practice and tested or verified through clinical interventions in treating diseases. Theoretical research is inseparable from the development of nursing education. Moreover, research methods should be specific and diverse. Further improvement of theories depends on patient-centered clinical practice. Therefore, this study plays an important role in the continuous enrichment and development of nursing theory, which is conducive to further promoting the progress of nursing management and nursing discipline.

## 1. Introduction

Nursing theory can function as a description, an explanation, guidance, or a prediction. Specifically, it can describe situations in nursing disciplines, explain relationships between phenomena, guide nursing practice, and predict nursing outcomes. Moreover, it may also provide a framework for analyzing and solving problems in nursing research, nursing management, nursing education, and clinical nursing [1]. Nursing theory has been developed for over a century, and relatively mature nursing theories, construction ideas, and methods have been established [2]. Bibliometrics visualize research results through literature analysis and bibliometric mapping to identify major trends in research development. As an effective tool for Big Data processing, bibliometrics has been widely applied in qualitative assessments of the development status and academic influence of a specific field [3]. However, no systematic bibliometric analysis of literature related to nursing theory has been conducted.

The Web of Science (WoS) database has adopted a set of strict selection procedures and an objective evaluation process. It includes the most authoritative and influential scholars, journals, and literature in various disciplines. Moreover, it clearly demonstrates a division of disciplines. Therefore, this study analyzed relevant literature on nursing theory research from the past 33 years, using the WoS database and the visualization analysis software VOSviewer, to understand the growth patterns, research hotspots, and development trends of nursing theory research publications worldwide, which can serve as a reference to promote clinical nursing theory research.

## 2. Data and Methods

### 2.1. Data Source

**Figure 1:**
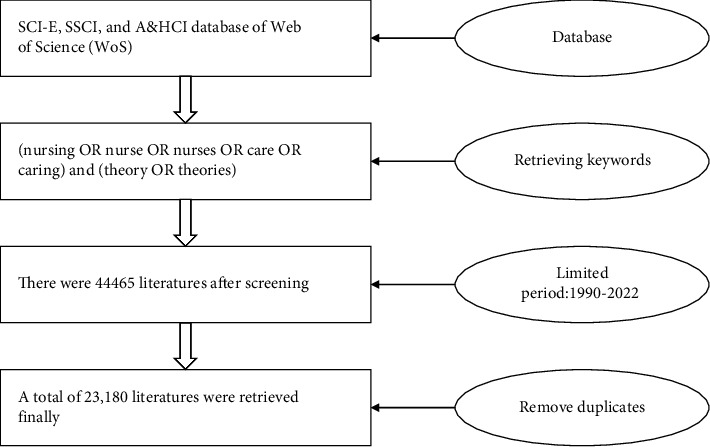
Flow chart of data collection.

WoS is the largest academic literature database covering the largest number of disciplines in the international academic community [4]. The journals included in the Science Citation Index Expanded (SCI-E) and Social Science Citation Index (SSCI) are mainly based on their impact factor (IF), which reflects the average citation frequency of journal articles. However, the journals included in the Arts and Humanities Citation Index (A&HCI) are based on the peer review results of major scholars in related disciplines worldwide. Therefore, the WoS core collection (SCI-E, SSCI, and A&HCI) database was selected, and an advanced search was performed. The search formula was set as TS = (nursing OR nurse OR nurses OR care OR caring) and TS = (theory OR theories); language type: English; document type: article & review; and time range: January 1, 1990–December 31, 2022 (retrieval date was January 6, 2022). In total, 44465 pieces of bibliographic information were downloaded. The downloaded bibliography was imported using NoteExpress software, and the duplicate content-checking function was used to remove duplicate documents. Incomplete bibliographies, character profiles, hospital or institution introductions, manuscript appointments, and documents with irrelevant research topics were eliminated after two investigators read the titles, abstracts, keywords, and other information, resulting in a final total of 23,180 documents. Data collection flow chart is shown in Figure 1.

### 2.2. Analysis Methods

The bibliometric analysis approach is based on bibliometrics theory and uses the literature information in the research field for analysis and investigation. This approach is currently widely applied in scientific research that uses existing literature as the research object. VOSviewer is a visualization tool for creating network diagrams that can perform quantitative analysis of literature and draw visual diagrams to reveal research hotspots and trends in a certain field. Each node in the visualization map represents a cited keyword, and the size of the node indicates the citation frequency. Connections between two nodes indicate the cocitation of a keyword, and the connection's thickness reflects the strength of that link. The distance between two nodes indicates a correlation in the co-occurrence network.

## 3. Results

### 3.1. Number of Publications and Cumulative Publications on Nursing Theory

**Figure 2:**
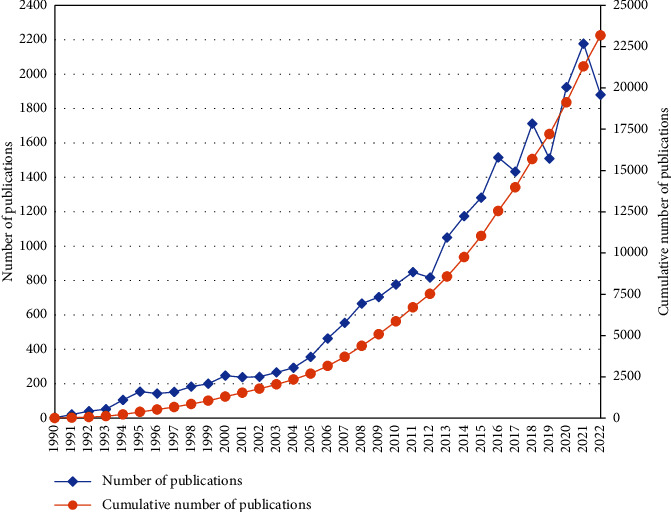
Number of publications and cumulative number of publications on nursing theory in the WoS database from 1990 to 2022.

In the past 33 years, 23,180 articles related to nursing theory have been published and included in the WoS database.  Figure 2 shows the general trend. In 1990, the database included only two publications, which increased to 154 articles by 1995, while the number of publications declined in 1996 and 1997. Compared with the number of publications in 1997, the number was higher from 1998 to 2011. During this period, the number of publications increased yearly until it reached 849 in 2011 and then fell again in 2012. In 2013, the number of publications gradually increased again, reaching its highest point in 2021, with an annual publication volume of 2177 articles and 1880 articles in 2022, which, despite being slightly lower than 2021, was still over twice the volume of ten years prior.

### 3.2. Distribution of Country (Region) of Nursing Theory Literature

A total of 130 countries (regions) had publications on nursing theory, with relatively concentrated distribution (Table 1), mainly in North America and Europe, such as the United States, the United Kingdom, Canada, and the Netherlands, as well as Australia. The United States provided 8,745 published articles, far exceeding other countries and accounting for 37.73% of the total publications. The United Kingdom, Canada, Australia, and the Netherlands accounted for approximately 13.95%, 8.81%, 7.56%, and 3.59% of the published articles, respectively. Regarding the number of published articles, the top five countries accounted for 71.64% of the total number of publications in nursing theory. A total of 808 articles on nursing theory were published in China (ranking sixth overall).

### 3.3. Subject Categories in Nursing Theory Publications

The published literature related to nursing theory included a total of 141 disciplines, with 12730 publications involving one discipline, 7983 publications involving two disciplines, 1710 publications involving three disciplines, and 99 publications involving four disciplines (Table 2). Among all the included disciplines, the top five were nursing, health care sciences and services, public, environmental, occupational health, psychology, and medicine general internal, as shown in Table 3.

### 3.4. Distribution of Institutions (Units) of Nursing Theory Publications

A total of 753 institutions (units) have published literature on nursing theory, and the top 30 institutions (units) and their cumulative number of publications are shown in Table 4. Most of the top 20 institutions in terms of publication volume were from the United States, the United Kingdom, Canada, and Australia. The University of Toronto and University of Alberta in Canada ranked highest, with 128 and 109 articles, respectively, followed by the University of Washington, University of North Carolina, University of California, San Francisco, and University of Michigan.

### 3.5. Journal Distribution of Nursing Theory Publications

The 23,180 research articles on nursing theory were published across 204 journals. The top 30 journals are listed in Table 5. The *Journal of Advanced Nursing* (IF 3.057) had the largest collection of nursing theory-related articles, with 985 articles published in 33 years. This was followed by the *Journal of Clinical Nursing*, *Nurse Education Today*, and *BMC Health Services Research*, each with collections of over 450 relevant publications, with IFs of 4.423, 3.906, and 2.908, respectively. According to Bradford's law [5] (i.e., if the journals are divided into three levels based on the number of publications on a certain subject within a certain period of time to make the number of relevant papers contained in each level equal, that is, exactly equal to one-third of the total number of articles on this subject published in all journals), it can be seen that the articles at the first level (core level) came from n1 journals, which are small in number but with the highest efficiency. After calculation, the core journals publishing articles on nursing theory were the top 30 journals, which included 7,766 related articles, accounting for approximately 33.50% of the total number of articles.

### 3.6. Distribution of Highly Productive Authors in Nursing Theory Publications

**Figure 3:**
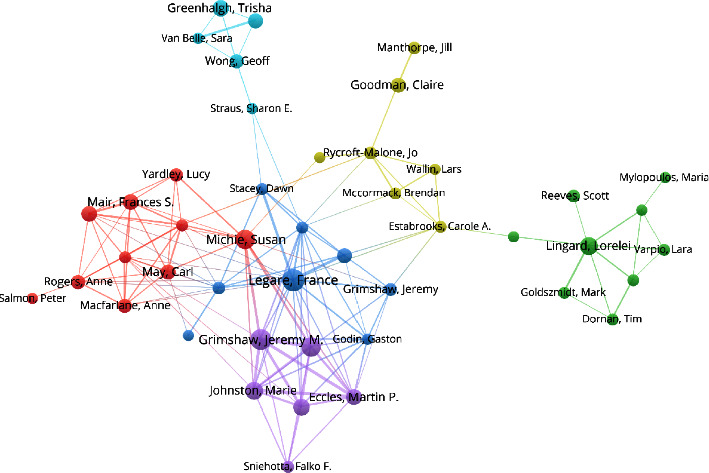
Collaboration network of highly productive authors of publications on nursing theory in WoS from 1990 to 2022.

A total of 57,853 authors were included in the publications on nursing theory, and Table 6 lists the top 20 most active authors. The most productive author of research articles on nursing theory was France Légaré from the Department of Family Medicine and Emergency Medicine, Laval University, Canada, who has published 31 related articles, followed by Jeremy M. Grimshaw from the Faculty of Medicine, University of Ottawa, Canada, who has published 28 articles, and Susan Michie from the Department of Clinical, Educational, and Health Psychology at the University College London; Marie Johnston from the Health Psychology Group at the University of Aberdeen; and Barbara Riegel from the School of Nursing at the University of Pennsylvania, who have all published more than 20 related articles. An analysis of cooperative research of the top 80 authors is shown in Figure 3. Most authors shared connections, indicating the establishment of cooperative relationships with a small number of noncollaborators around the cooperative network [6]. The closeness of the cooperative relationships between authors can be seen from the network density. The top ten most highly productive authors all had collaborators, indicating the vital importance of cooperation between researchers.

### 3.7. Top 10 Funding Organizations for Publications on Nursing Theory

Scientific research funds may significantly promote the development of health care, and the funded research articles may reflect the latest scientific research level to a certain extent and are highly informative documents. The science funding system has become a strong driving force for the rapid development of nursing research, has produced excellent output, and has contributed greatly to scientific research articles [7]. Among the 23,180 publications related to nursing theory, 18,966 were funded, involving 197 funding categories in total.  Table 7 shows the top ten funding agencies. The US Department of Health and Human Services funded the largest number of nursing theory publications, followed by the US National Institutes of Health, Canadian Institutes of Health Research, National Natural Science Foundation of China, and UK National Institute for Health and Care Research.

### 3.8. Co-Occurrence Analysis and Coword Clustering Analysis of High-Frequency Keywords in Nursing Theory Publications

**Figure 4:**
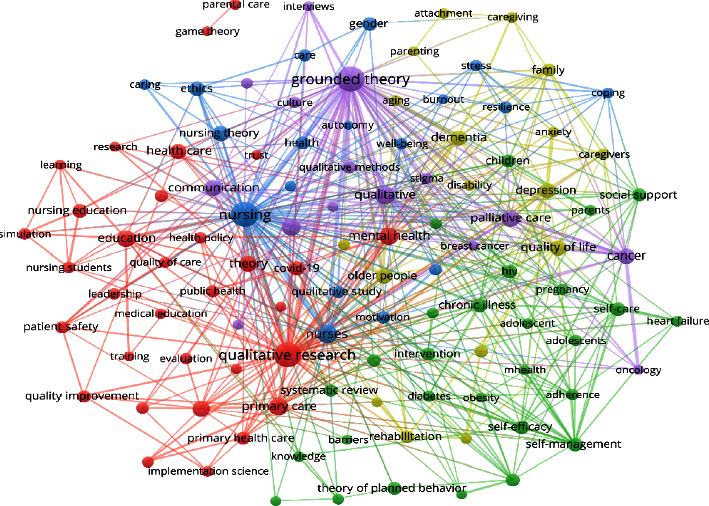
Co-occurrence network of high-frequency keywords in nursing theory publications in WoS from 1990 to 2022.

Coword clustering is a multivariate statistical method that measures the relationships between data and provides classifications based on similarities between index data. According to bibliometrics principles, no uniform standards are placed on the number of high-frequency words in coword clustering analysis. Should the number of high-frequency words be too small, it would be unable to reflect the structure of the subject. Conversely, a selection range that is too large would interfere with the analysis. At present, words with a cumulative frequency reaching approximately 40% of the total frequency are generally selected as high-frequency words [8]. In this study, 23,180 nursing theory-related publications in 33 years included a total of 47,365 keywords. After thorough consideration of expert advice and multiple adjustments, keywords with a frequency greater than or equal to 80 were selected (125 keywords) to plot the co-occurrence network of high-frequency keywords in nursing theory publications from 1990 to 2022 found on WoS (Figure 4). The research hotspots were summarized based on specific literature content, co-occurrence of high-frequency keywords, teaching experience, clinical experience, research experience, and the software's clustering function to ultimately obtain seven hotspots in nursing theory research from 1990 to 2022 as follows: primary health care, psychological ethics, social support, nursing intervention, nursing education and research, older people and chronic diseases, and quality of life (Table 8).

### 3.9. Topic Evolution of Nursing Theory Publications

#### 3.9.1. 1990–2000

**Figure 5:**
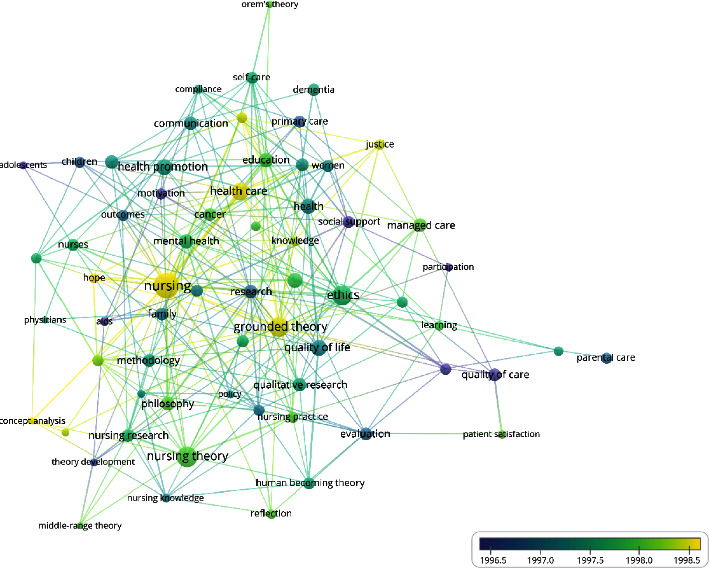
Co-occurrence network of high-frequency keywords in nursing theory publications in WoS from 1990 to 2000.

A total of 4047 keywords were identified. In this study, keywords (65) with a frequency greater than or equal to seven were selected to plot the co-occurrence network of high-frequency keywords in nursing theory publications in WoS from 1990 to 2000 (Figure 5). The research hotspots were summarized based on specific literature content, co-occurrence of high-frequency keywords, teaching experience, clinical experience, research experience, and the software's clustering function to ultimately obtain 10 hotspots of nursing theory research from 1990 to 2022 as follows: psychology, ethics, family nursing, nursing outcomes, quality of life, primary health care, children and adolescents, nursing research, theoretical models, and cancer and chronic diseases (Table 9).

#### 3.9.2. 2001–2011

**Figure 6:**
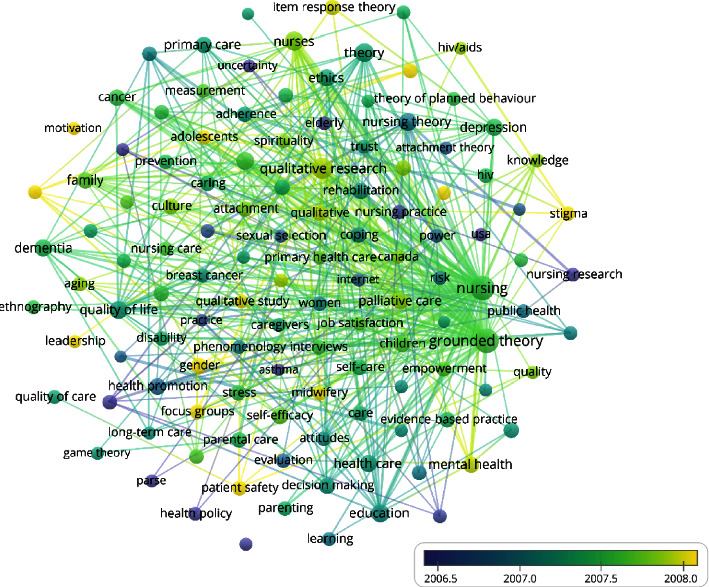
Co-occurrence network of high-frequency keywords in nursing theory publications in WoS from 2001 to 2011.

A total of 14,367 keywords were identified. In this study, keywords (135) with a frequency greater than or equal to 20 were selected to plot the co-occurrence network of high-frequency keywords in nursing theory publications in WoS from 2001 to 2011 (Figure 6). The research hotspots were summarized based on specific literature content, co-occurrence of high-frequency keywords, teaching experience, clinical experience, research experience, and the software's clustering function to ultimately obtain 15 hotspots of nursing theory research from 2001 to 2011 as follows: health promotion, nurse decision-making, evidence-based practice, methodology, long-term care, parental care, women's health, home-based care, hospice care, older people, sexually-transmitted diseases (STDs), qualitative research, assessment, nursing practice, and philosophy (Table 10).

#### 3.9.3. 2012–2022

**Figure 7:**
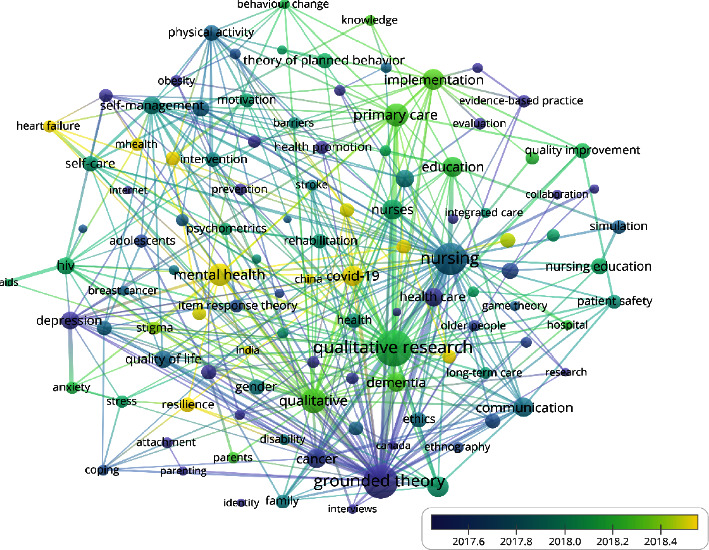
Co-occurrence network of high-frequency keywords in nursing theory publications in WoS from 2012 to 2022.

A total of 30,457 keywords were identified. In this study, keywords (121) with a frequency greater than or equal to 40 were selected to plot the co-occurrence network of high-frequency keywords of nursing theory publications in WoS from 2012 to 2022 (Figure 7). The research hotspots were summarized based on specific literature content, co-occurrence of high-frequency keywords, teaching experience, clinical experience, research experience, and the software's clustering function to ultimately obtain eight hotspots of nursing theory research from 2012 to 2022 as follows: patient-centered, grounded theory, psychological ethics, nursing education, nursing intervention, nursing theory, quality of life, and aging (Table 11).

## 4. Discussion

### 4.1. International Research on Nursing Theory Has Been Active

Nursing is an interdisciplinary subject [9]. The number of publications related to nursing theory has shown an annual increase in recent years, with nearly 10,000 nursing theory publications involving more than two disciplines. The top eight journals with the highest number of publications are the *Journal of Advanced Nursing* (IF 3.057), *Journal of Clinical Nursing* (IF 4.423), *Nurse Education Today* (IF 3.906), *BMC Health Services Research* (IF 2.908), *Social Science & Medicine* (IF 5.379), *Nursing Science Quarterly* (IF 0.833), *Qualitative Health Research* (IF 4.233), and *BMJ Open* (IF 3.007), with a cumulative percentage of 19.38%. The latest research findings of nursing theory may be learned and drawn on by paying more attention to these journals.

### 4.2. The United States Has the Leading Nursing Theory Research Globally

Foreign research institutions with publications on nursing theory were mainly found to be in economically developed countries, such as the United States and some European countries. Over 90% of the top 20 most highly productive authors were from universities and affiliated teaching hospitals. Over one-third of the foreign nursing theory research articles were from the United States. These publications received the most funding from the United States Health and Human Services Department and the National Institutes of Health, showing that the nursing theory research in the United States is at the forefront of the world, leading the developmental trends in nursing theory today.

### 4.3. Cooperative Research Will Be the Trend for Future Nursing Theory Studies

Scientific research cooperation is an effort that can significantly improve scientific research capacity without much investment and has become the mainstream method of social science research today. This study's results show that highly productive authors generally have collaborators, indicating the importance of cooperation among researchers.

### 4.4. Cluster Analysis of High-Frequency Keywords Revealed That Nursing Theory Research Tends toward Focusing on Clinical Application and Verification

Keywords are the natural language that reflects the core content of research. The more keywords appear in a document, the more the main research content can be represented. The more the number of co-occurrences of two high-frequency keywords, the stronger the association between the two. After clustering, the keywords may further reflect the focus of the research field and the academic topics in which researchers are interested.

We summarized the research hotspots based on the specific literature content and keyword cluster analysis. Finally, seven hot categories of nursing theory research from 1990 to 2022 formed, including primary health care, psychological ethics, social support, nursing intervention, nursing education and research, older people and chronic diseases, and quality of life. Nursing theory research has also focused on psychological ethics and social support on top of meeting patients' most basic primary health care requirements. There is more and more research on nursing theories, and a variety of nursing theories have been formed, such as Oram's self-care theory, Erickson's psychosocial development theory, and Piaget's cognitive development theory, which effectively guide clinical nursing practice and have been verified and tested in the process of clinical nursing practice. In the 21st century, many countries have become aging societies, and nursing theory has also often been applied in the care of older people and chronic diseases to improve patients' quality of life. Theoretical research is inseparable from the development of nursing education. Further improvements in theory will require more research based on clinical practice.

From 1990 to 2000, there were 10 research hotspots related to nursing theory, including psychology, ethics, family care, care outcomes, quality of life, primary health care, adolescents, nursing research, theoretical models, and cancer chronic diseases. From 2001 to 2012, nursing theory research formed fifteen research hotspots, including health promotion, nurse decision-making, evidence-based practice, methodology, long-term care, parental care, women's health, home care, hospice care, the elderly, sexually transmitted diseases, qualitative research, assessment, nursing practice, and philosophy. From 2013 to 2022, eight research hotspots related to nursing theory have been formed, including patient-centered, grounded theory, psychological ethics, nursing education, nursing intervention, nursing theory, quality of life, and aging. In terms of the research object, theoretical research has evolved from children and adolescents at the beginning to women's health and has recently moved its focus to application among older adults. In terms of research content, theoretical research has changed from family or home-based care to patient-centered clinical care. Regarding nursing outcomes, theoretical research has changed from patient safety and health promotion to improving the quality of life. Research methods have changed from concept analysis at first to qualitative research and specific, grounded theories. In terms of other aspects, given the convenience of the Internet and mobile data acquisition today, nursing theory research has become increasingly concerned with the actual needs of patients. It aims to change the behaviors of nurses and patients to promote health through evidence-based, theory-guided interventions.

## 5. Conclusions

In summary, bibliometric methods and VOSViewer software were used in this study to analyze the literature in English on nursing theory included in the WoS database during the 33 years between 1990 and 2022. Historical changes in nursing theory were explored based on the annual number of publications, and the authoritative academic journals of nursing theory were identified based on the number of articles published. Moreover, the leaders of the theoretical discipline and their academic research teams were identified according to the authors and institutions. Moreover, the research hotspots and developmental trends of nursing theory were explored based on keyword clustering and topic evolution analysis. Nursing theory research has been increasingly applied to clinical practice and tested or verified through disease intervention. The theoretical research is inseparable from the development of nursing education. The methodologies of theoretical studies should be specific and diversified. Further improvement in nursing theory requires patient-centered clinical practice [10]. Therefore, according to the suggestions and guidance of nursing theory and nursing practice in this study, the advancement of this study will be very conducive to improving and enhancing nursing management and thus has vital guiding significance for the nursing discipline.

## Figures and Tables

**Table 1:** List of the top 30 countries (regions) in terms of the cumulative number of publications in WoS from 1990 to 2022.

No	Country	Documents
1	USA	8745
2	England	2681
3	Canada	2043
4	Australia	1753
5	Netherlands	832
6	China	808
7	Sweden	465
8	Norway	442
9	Scotland	382
10	Germany	376
11	Singapore	301
12	Spain	255
13	Denmark	227
14	Brazil	202
15	Italy	195
16	France	177
17	Israel	165
18	Finland	149
19	Belgium	145
20	South Africa	133
21	Japan	129
22	Ireland	124
23	New Zealand	120
24	Iran	117
25	Wales	105
26	South Korea	104
27	Switzerland	104
28	India	87
29	Turkey	77
30	Northern Ireland	66

**Table 2:** Subject categories involved in WoS publications from 1990 to 2022.

No	Subject category	Documents
1	4	99
2	3	1710
3	2	7983
4	1	12730

**Table 3:** Top 30 disciplines involved in WoS publications from 1990 to 2022.

No	Subject	Documents
1	Nursing	4016
2	Health care sciences & services	2031
3	Public, environmental, & occupational health	1939
4	Psychology	1526
5	Medicine general internal	1129
6	Education & educational research	926
7	Geriatrics & gerontology	883
8	Social sciences-other topics	784
9	Business & economics	693
10	Computer science	643
11	Psychiatry	523
12	Family studies	491
13	Environmental sciences & ecology	437
14	Oncology	406
15	Information science & library science	323
16	Social work	301
17	Engineering	251
18	Neurosciences & neurology	246
19	Rehabilitation	231
20	Behavioral sciences	204
21	Pediatrics	173
22	Sociology	165
23	Dentistry, oral surgery, & medicine	145
24	Life sciences & biomedicine-other topics	137
25	Obstetrics & gynecology	116
26	Chemistry	95
27	Research & experimental medicine	93
28	Communication	89
29	Cardiovascular system & cardiology	88
30	Anthropology	85

**Table 4:** List of the top 30 institutions (units) of the cumulative number of publications in WoS from 1990 to 2022.

No	Organization	Country	Documents
1	University of Toronto	Canada	128
2	University of Alberta	Canada	109
3	University of Washington	United States of America	108
5	University of North Carolina	United States of America	104
6	University of California, San Francisco	United States of America	103
7	University of Michigan	United States of America	99
8	Monash University	Australia	98
9	University of Wisconsin	United States of America	95
10	University of Pennsylvania	United States of America	94
11	University of Illinois	United States of America	92
12	Karolinska Institute	Sweden	89
13	University of Sydney	Australia	85
14	University of British Columbia	Canada	83
15	King's College London	United Kingdom	80
16	McMaster University	United Kingdom	79
17	University of Missouri	United States of America	75
18	University of California, Los Angeles	United States of America	74
19	University of Nottingham	United Kingdom	71
20	University of Gothenburg	Sweden	70
21	University of Sheffield	United Kingdom	67
22	University of Minnesota	United States of America	65
23	University of Oslo	Norway	61
24	Linkoping University	Sweden	59
25	Columbia University	United States of America	58
26	University of Melbourne	Australia	57
27	University of Sao Paulo	Brazil	56
28	Boston University	United States of America	55
29	University of Queensland	Australia	54
30	McGill University	Canada	52

**Table 5:** Top 30 journals in terms of the cumulative number of publications in WoS from 1990 to 2022.

No	Journal	Publications
1	Journal of advanced nursing	1202
2	Journal of clinical nursing	588
3	Nurse education today	544
4	BMC health services research	514
5	Social science & medicine	506
6	Nursing science quarterly	415
7	Qualitative health research	362
8	BMJ open	361
9	Advances in nursing science	276
10	International journal of nursing studies	225
11	Scandinavian journal of caring sciences	204
12	Implementation science	186
13	Journal of evaluation in clinical practice	170
14	Midwifery	161
15	Journal of medical internet research	158
16	BMC public health	153
17	Nursing ethics	147
18	Patient education and counseling	145
19	PLOS one	140
20	Children and youth services review	132
21	Journal of nursing management	129
22	Revista latino-americana de enfermagem	128
23	Nursing inquiry	123
24	Journal of general internal medicine	120
25	Cancer nursing	117
26	British journal of social work	115
27	Journal of interprofessional care	114
28	Academic medicine	113
29	Journal of nursing scholarship	111
30	Revista da escola de enfermagem da usp	107

**Table 6:** Top 20 highly productive authors in terms of the cumulative number of publications in WoS from 1990 to 2022.

No	Author	Institution	Publications
1	Légaré, France	Department of Family Medicine and Emergency Medicine, Laval University, 1050, Avenue de la Médecine, Québec, QC, G1V 0A6 Canada	31
2	Grimshaw, Jeremy M.	Centre for Implementation Research, Ottawa Hospital Research Institute - General Campus, Ottawa, Ontario, Canada; Faculty of Medicine, University of Ottawa, Ottawa, Ontario, Canada	28
3	Francis, Jill J.	Centre for Implementation Research, Ottawa Hospital Research Institute - General Campus, Ottawa, Ontario, Canada; School of Health Sciences, University of Melbourne, Melbourne, Victoria, Australia	25
4	Michie, Susan	Department of Clinical, Educational and Health Psychology, Centre for Behaviour Change, University College London, London, WC1E 7HB, UK	24
5	Johnston, Marie	Health Psychology Group, University of Aberdeen, Aberdeen, AB25 2ZD, Scotland, UK	21
6	Lingard, Lorelei	Centre for Education Research & Innovation, Schulich School of Medicine & Dentistry, and Professor Faculty of Education, Western University, London, Ontario, Canada	21
7	Riegel, Barbara	School of Nursing, University of Pennsylvania, Philadelphia	20
8	Greenhalgh, Trisha	Nuffield Department of Primary Care Health Sciences, University of Oxford, Radcliffe Primary Care Building, Radcliffe Observatory Quarter, Oxford, UK	18
9	Presseau, Justin	Centre for Implementation Research, Ottawa Hospital Research Institute, Ottawa, Canada; School of Epidemiology & Public Health, University of Ottawa, Ottawa, Canada; School of Psychology, University of Ottawa, Ottawa, Canada	17
10	Eccles, Martin P.	Institute of Health and Society, Newcastle University, Baddiley-Clark Building, Richardson Road, Newcastle Upon Tyne, NE2 4AX, UK	17
11	Vellone, Ercole	Department of Biomedicine and Prevention, University of Rome Tor Vergata, Italy	17
12	Braithwaite, Jeffrey	Australian Institute of Health Innovation, Macquarie University, Sydney, NSW, Australia	17
13	Mair, Frances S.	General Practice and Primary Care, Institute of Health and Wellbeing, University of Glasgow, Glasgow, GB, UK	16
14	May, Carl R.	Department of Health Services Research and Policy, Faculty of Public Health and Policy, London School of Hygiene and Tropical Medicine, London, United Kingdom	15
15	Granek, Leeat	School of Health Policy and Management and Department of Psychology, Faculty of Health, York University, Toronto, Ontario, Canada	15
16	Ni, Pengsheng	Department of Health Law, Policy, and Management, School of Public Health, Boston University, Boston, MA	14
17	Gagnon, Marie-Pierre	Faculty of Nursing, Université Laval, Québec, Canada	14
18	Marchal, Bruno	Department of Public Health, Institute of Tropical Medicine, Antwerpen, Belgium	14
19	Goodman, Claire	Centre for Research in Public Health and Community Care, university of Hertfordshire, Hatfield, United Kingdom; NIHR Applied Research Collaboration-East of England (ARC-EoE), Cambridge, United Kingdom	14
20	Williams, Geoffrey C.	Department of Clinical and Social Sciences in Psychology, University of Rochester, Rochester, NY, USA	13

**Table 7:** Top 10 funding agencies from which publications on nursing theory in WoS from 1990 to 2022 received funding.

No	Funding	Publications
1	United States Department of Health Human Services	1935
2	National Institutes of Health (NIH)	1526
3	Canadian Institutes of Health Research (CIHR)	327
4	National Natural Science Foundation of China	276
5	National Institute for Health Research (NIHR)	235
6	National Institute of Mental Health	214
7	Economic Social Research Council (ESRC)	209
8	Agency for Healthcare Research Quality	207
9	National Science Foundation (NSF)	203
10	European Commission	201

**Table 8:** List of research hotspots in the clustering of high-frequency keywords in nursing theory publications in WoS from 1990 to 2022.

Clustering	Research hotspot	Keywords
1	Primary healthcare	Primary care, health policy, healthcare, implementation, ethnography, leadership, evidence-based practice, evaluation, patient safety, simulation, quality of care, quality improvement, primary health care
2	Psychological ethics	HIV/AIDS, COVID-19, parenting, ethics, attachment, health, depression, stress, spirituality, well-being, mental health, autonomy, risk, decision-making, gender, resilience
3	Social support	Stigma, culture, women, parental care, social support, disability, adolescents, children, Canada, barriers
4	Nursing intervention	Motivation, theory of planned behavior, self-efficacy, self-management, knowledge, diabetes, chronic illness, self-determination theory, health promotion, attitudes, prevention, intervention, physical activity, self-care, intervention, adherence
5	Nursing education and research	Nursing, nursing education, qualitative research, nursing practice, concept analysis, grounded theory, philosophy, theory, power, focus groups, public health, phenomenology, grounded theory, game theory, empowerment, learning, Australia, nursing research, nursing theory, recovery
6	Older people and chronic diseases	Communication, cancer, caregivers, dementia, older people, coping, caregiving, dementia, palliative care, long-term care, communication, family, interviews,
7	Quality of life	Quality of life, rehabilitation, job satisfaction, nurses, nurses, item response theory, anxiety

**Table 9:** List of research hotspots in the clustering of high-frequency keywords in nursing theory publications in WoS from 1990 to 2000.

Clustering	Research hotspot	Keywords
1	Psychology	Mental health, social support, decision making, depression, compliance, motivation
2	Ethics	Ethics, sexual selection, AIDS, justice
3	Family nursing	Communication, family, coping, self-care, women, knowledge
4	Nursing outcome	Nursing, quality of care, patient satisfaction, education, nursing knowledge, outcomes, reflection, nursing practice, managed care
5	Quality of life	Hope, participation, quality of life, physicians, doctor-patient relationship, empowerment, nurses
6	Primary healthcare	Culture, health policy, health care, primary health care, economic evaluation, health, health promotion, primary care, policy
7	Children and adolescents	Learning, adolescents, children, pregnancy, parental care
8	Nursing research	Phenomenology, grounded theory, methodology, nursing research, evaluation, qualitative research, epistemology, concept analysis, philosophy, research
9	Theoretical model	Theory-practice gap, nursing theory, models, parse theory, human becoming theory, theory of planned behavior, systems theory
10	Cancer chronic diseases	Chronic illness, cancer, dementia

**Table 10:** List of research hotspots in the clustering of high-frequency keywords in nursing theory publications in WoS from 2001 to 2011.

Clustering	Research hotspot	Keywords
1	Health promotion	Health, depression, autonomy, health promotion, risk, health care, breast cancer, prevention, UK, trust, compliance, adherence, culture, phenomenology
2	Nurse decision-making	Motivation, health policy, communication, learning, evaluation, decision-making, nurse education, intervention, primary health care
3	Evidence-based practice	Self-efficacy, interaction, midwifery, pregnancy, attachment, interviews, primary care, leadership, evidence-based medicine, attachment theory, family nursing, focus groups, evidence-based practice, parenting, family
4	Methodology	Education, decision making, ethics, uncertainty, bioethics, rehabilitation, USA, methodology
5	Long-term care	Dementia, power, job satisfaction, critical theory, long-term care, older people, Internet, empowerment
6	Parental care	Parental care, Canada, game theory, quality of care, sexual selection
7	Women's health	Women, diabetes, women's health, research, children, attitudes, practice, knowledge, adolescents, self-care, pain, patient safety, disability, stigma
8	Home-based care	Nursing care, nurses, managed care, concept analysis, nursing, home care, quality, mental health, chronic illness, nursing education, ethnography
9	Hospice care	Hope, palliative care, cancer, coping, stress, patient education, measurement, aging, asthma, spirituality
10	Elderly	Elderly, care, caregiving, grounded theory, caring, aged
11	STD	HIV/AIDS, gender
12	Qualitative research	Qualitative research, social support, theory development, qualitative, qualitative methods
13	Assessment	Caregivers, validity, reliability, assessment
14	Nursing practice	Theory, theory of planned behavior, nursing practice, nursing research, nursing theory, item response theory, parse
15	Philosophy	Public health, quality of life, philosophy

**Table 11:** List of research hotspots in the clustering of high-frequency keywords in nursing theory publications in WoS from 2012 to 2022.

Clustering	Research hotspot	Keywords
1	Patient-centered	Knowledge translation, patient safety, chronic illness, primary care, patient-centered care, technology, public health, healthcare, implementation, hospital, ethnography, leadership, integrated care, evaluation, social support, primary care, evidence-based practice, risk, health policy, older people, quality improvement, process evaluation
2	Grounded theory	Communication, cancer, qualitative research, family, grounded theory, qualitative, coping, palliative care, family, adolescents, children, attachment, decision-making, qualitative methods, decision-making, interviews, identity, resilience
3	Psychological ethics	HIV/AIDS, COVID-19, stigma, stress, pregnancy, mental health, India, gender, mental health, women, barriers, South Africa, Canada, health disparities
4	Nursing education	Nursing education, education, nursing, nurse, well-being, medical education, nursing students, medical education, Australia, simulation, China, assessment, job satisfaction
5	Nursing intervention	Adherence, exercise, knowledge, health, health promotion, attitudes, motivation, behavior change, self-efficacy, prevention, self-care, self-management, Internet, intervention, physical activity, heart failure, diabetes, intervention, prevention, obesity
6	Nursing theory	Ethics, nursing practice, concept analysis, theory of planned behavior, self-determination theory, game theory, health, spirituality, culture, nursing theory, item response theory, theory, research, phenomenology, item response theory, autonomy
7	Quality of life	Breast cancer, quality of life, rehabilitation, older adults, dementia, disability, depression, quality of care, stroke, long-term care, psychometrics, anxiety
8	Aging	Parenting, parental care, decision-making, aging, parents, recovery

## Data Availability

The data used can be found in the references listed.
